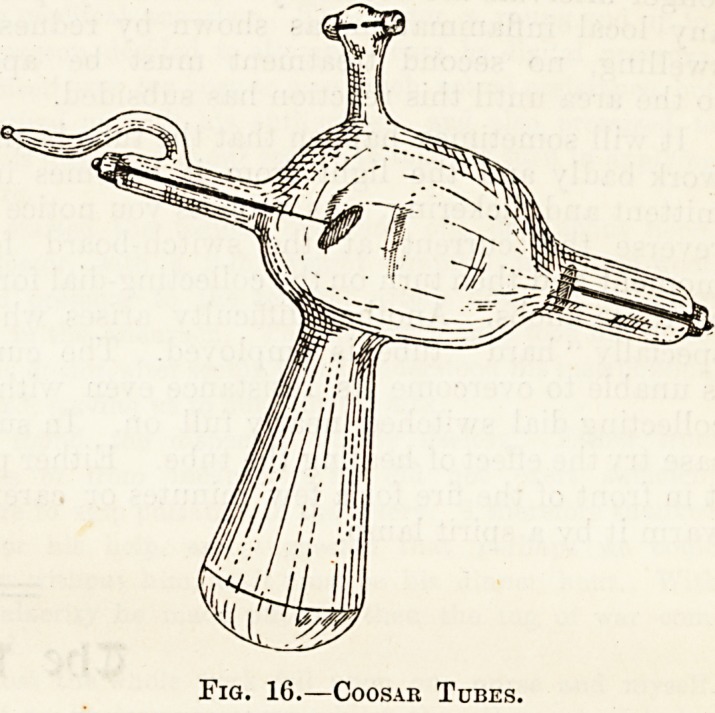# The Hospital. Nursing Section

**Published:** 1905-09-16

**Authors:** 


### The Hospital.

### Nursing Section.

Contributions for this Section of "The Hospital" should be addressed to the Editor, "The Hospital" Nursing Section, 28 & 29 Southampton Street, Strand, London, W.C.

No. 990.—Vol. XXXVIII. SATURDAY, SEPTEMBER 16, 1905.

### Notes on News from the Nursing World.

### OUR CHRISTMAS DISTRIBUTION.

This year we have purposely postponed reminding our readers of our Christmas Distribution until most of them, at any rate, have come back from their holidays invigorated and prepared to undertake a little extra work for the sake of helping those whom they know, from their own personal experience, to be badly in want of assistance. The fact that they have been fortunate enough to enjoy a happy and health-giving holiday themselves at the seaside, in the country, or abroad, will, we hope and believe, supply an incentive to them to send us at least one garment as a thankoffering. This can be done by the practice of a little self-denial, and we are sure that the kind-hearted nurses who are so often grieved because of the needs of hospital and infirmary patients in respect to even necessary articles of clothing, will not grudge the few hours they spend in responding to our appeal. Of course, large parcels are very acceptable, but we should like this year to be able, in addition to these, to announce a record list of contributions of single garments. They should, in all cases, be addressed to the Editor, 28 & 29 Southampton Street, Strand, London, W.C., with "Clothing Distribution" marked on the outside.

### POOR-LAW NURSING AND THE SELECT COMMITTEE.

At the meeting of the Council of the Association of Poor-law Unions of England and Wales last week, Dr. Rhodes, of Chorlton, complained that not a single witness who knew anything about the Poor Law had given evidence before the Select Committee on the Registration of Nurses. He proposed that immediate steps should be taken in the matter, and the proposal was agreed to. But this is a day after the fair. It is not the case that all the witnesses who gave evidence before the Select Committee were ignorant of the Poor Law; and, while we admit that those who are specially interested in Poor-law nursing were not adequately represented at the sittings of the Committee, we are afraid that Dr. Rhodes and his friends have only themselves to blame in the matter. So far as we know the Committee were quite prepared to hear evidence from every quarter which was calculated to assist them in their inquiry. It is not too late, however, if legislation on the lines of the report be attempted, for authorities on the Poor Law to obtain a hearing.

### CHARACTER AND REGISTRATION.

On the strength of the fact that the report of the Select Committee on the Registration of Nurses mentions a few instances of women who are a disgrace to the profession, publicity is afforded in a Glasgow paper to a long article, with head-lines of a sensational character, purporting to be written by a surgery dispenser, the substance of which is that many nurses are addicted to tippling. One of the sensational head-lines, is, in fact, "Ministering Angels and the Bottle." Having quoted the cases referred to in the report of the Select Committee, the writer goes on to make sweeping charges against Glasgow nurses, and avers that there are, in that city, "scores and scores of women who ought never to be in the nursing profession." In support of this damaging allegation two illustrations are given. Of course, a woman who has a weakness for stimulants is utterly unfit to be a nurse; but the contention of the author of the indictment which appears in the columns of our Glasgow contemporary, that State Registration would put an end to tippling nurses, shows the lengths to which the supporters of that panacea are driven in order to advocate it. Surely, it is obvious that the possession of a certificate from the best of schools is no guarantee that the owner is devoid of the infirmities of human nature. Character, which is even more essential than training, cannot be ensured by any system of registration.

### THE NURSE-STEWARDESS.

There is an air of mystery enveloping the relations of the Royal Mail Steam Packet Company and their nurses, at any rate so far as the head office is concerned. Our commissioner, whose account of his search for information is given in another column, was told that details are obtainable at Southampton. We should have thought that they might have been given in London. But there is an explanation for this reticence which we fully appreciate. The company is inundated with applications for appointments, and it is obviously a saving of time and trouble to refer all callers and correspondents to Southampton. It is clear, however, that the woman who undertakes the position of nurse-stewardess has to sink the nurse in the stewardess, except in emergencies, when her training is of course highly valuable. But those who imagine that a nurse-stewardess on a liner ranks as a nursing sister does in the Army are under an erroneous impression. She does not mess with the passengers, nor, if there are social functions on board, will she find that she needs an evening costume. For a nurse who does not mind what work she does, is fond of the water, likes to see foreign countries, and, colloquially speaking, can "rough it," the experience, if she can get it may be worth having.

### NURSING AND THE CHURCH.

We lately noticed the fact that Miss Amy Hughes is to read a paper on nursing at the Church Congress next month, and now we observe that at the Carlisle Diocesan Conference on Tuesday and Wednesday next Dr. Henry Barnes is to speak on "Town and Country Nursing." It is satisfactory to find that the question of nursing is coming to the front at meetings in connection with the Church. Its importance from the Church point of view cannot easily be over-estimated, and the more freely it is discussed when diocesan or other Church bodies meet together the better, so long as no attempt is made to associate it with any particular theological bias.

### SISTERS' SALARIES AND EVENING FROCKS.

The matron of the Royal South Hants and Southampton Hospital states that one of her sisters came to her the other day, and, referring to her salary, which she wished to be increased, said, "You see, matron, it does not even run to a new evening frock." Miss Mollett agreed, but she had the courage to express the doubt whether charitable institutions, supported by voluntary subscriptions, have any right to increase their sisters' salaries to allow for the provision of sufficient evening frocks. The labourer is worthy of her hire, and hospital sisters are entitled to expect sufficient salaries for their responsible duties. There is no reason why a nurse should not wear an evening frock when she is among her friends, but we do not think that a committee, who act as the stewards of the charitable public, would be warranted in raising the scale of remuneration to the nursing staff for the purpose of enabling its members to indulge in harmless and becoming luxuries.

### THE AUTOCRAT OF THE ISOLATION HOSPITAL.

Last week we drew attention to a flagrant instance of maladministration of an isolation hospital. This week a correspondent, who has lately been appointed matron of another of these institutions, describes a state of affairs, which, if less serious, is not less exasperating. Apparently, the committee of the latter allow the porter to have his own way, which is by no means sweet, and the former matron left because she could not get on comfortably with the autocrat of the isolation hospital. The Corporation under whom he is allowed to exercise sway, consists of several excellent citizens. Cannot they see that it is their duty to their new matron to take care that she has a free hand, and is in no respect troubled by the porter?

### BRADFORD DOCTORS ON NURSES.

At the distribution of prizes to the nurses of the Bradford Royal Infirmary last week, the gold medal was awarded to Nurse Harborough, the silver medals to Nurses Denison and Jameson, and book prizes for bandaging work to Nurses Bibby and Fawthorp. After the ceremony one of the medical men attached to the infirmary addressed some remarks to the nurses in training, urging them specially to guard against what he called flippancy. For nurses to speak in a trivial way of matters of seriousness he considered lowered and degraded them in the eyes of those who heard them. Another doctor, alluding to the question whether the nurses are over-trained, said that there was no doubt that some nurses, when leaving hospital, assumed a certain pride of knowledge and imagined that they were in a position to lay down the law in respect to medical matters. The consequence was that nurses were not welcome in some homes. We think that the nurse who invests herself with undue importance is not only unwelcome in some homes, but can hardly be welcome in any. In private nursing it is the helpful rather than the clever nurse who wins golden opinions.

### NURSES AND WASTE.

It is asserted in the columns of a contemporary by a correspondent, who, alluding to an article which recently appeared in The Hospital, affirms that there is "no class that waste more wantonly or more wilfully than nurses." This is one of those sweeping accusations which it is very easy to make and very hard to meet. That nurses do not always exercise economy is undoubtedly true, but this also applies to most other classes; and though they may be occasionally wasteful, a temptation to which they are often subjected we think that their wastefulness is usually due to thoughtlessness rather than to wilfulness. Those who level wholesale charges against them should, at any rate, be able to cite individual cases of wanton and wilful waste. Unless and until they do so, we do not think that fair-minded people will be at all disposed to treat such allegations seriously.

### IN THE SIXTIES.

In relating his experiences of his forty years' service as clerk to the Toxteth Guardians, Mr. James Moulding, one of the best known of Poor-law officers in the north of England, mentions a debate which took place in the sixties on a proposal to appoint a second nurse to the workhouse infirmary. The inmates then numbered about 350, as compared with 1,200 for whom accommodation is provided to-day. Mr. Moulding says: "The suggestion that a second nurse should be engaged was looked upon as a piece of extravagance on the part of certain Guardians, and elicited a really exciting and protracted discussion, which nowadays would afford much amusement, especially when it is recollected that our nursing staff in the workhouse to-day numbers 42." Whatever improvements may be still essential with regard to workhouse nursing generally, it must be admitted that the progress which Mr. Moulding has seen at Toxteth is by no means exceptional.

### THE MATRON OF LIMERICK COUNTY INFIRMARY.

In connection with the eleventh anniversary of Miss Mayne's appointment as matron of the County Infirmary, Limerick, which has just been celebrated, a pleasing function took place in the shape of the presentation of a handsome gold watch and chain. Miss Mayne was trained at Dr. Steevens' Hospital, Dublin, and some of her old friends in this institution joined in the presentation with her friends in Limerick. The ceremony took place at Mountjoy Private Hospital, Dublin, and Miss Kelly, lady superintendent of Dr. Steevens' Hospital, in handing the watch to the matron, referred to her great skill as a nurse and to her popularity with her patients and co-workers. Several well-known members of the nursing profession were present on the occasion. The presentation must be all the more acceptable to Miss Mayne at a time when she has had to put up with a great deal of unjust criticism, and to contend with difficulties which ought not to have been thrown in her way.

### FALSE ECONOMY AT WINDSOR.

We regret to learn that, on further consideration, the Windsor Guardians have finally determined to ignore the views of the medical officer and the head nurse. Instead of acting upon their advice to fill a vacancy on the staff by the selection of a fully-trained nurse, they decided, at their last meeting, to advertise for an assistant nurse only. No other reason can be assigned for this course than that the salary of an assistant nurse, who is not, of course, fully trained, is less than that of a qualified person. But the policy pursued by the Windsor Guardians, in face of the recommendations of the experts in their employ, is one of false economy, because no economy which is not consistent with efficiency can be otherwise.

### AN ARGYLL ASSOCIATION IN DIFFICULTIES.

At the annual meeting of the North Argyll Nursing Association, the secretary reported that during the past year the six nurses in the service of the organisation had paid 2,819 visits to 261 patients. The income for the twelve months was £274, and the expenditure £318. It was also announced that two districts had withdrawn from the association in order to join the Argyllshire County Nursing Association. The prospect is not, therefore, promising, and a reduction in the number of nurses, who judging by the figures cited are rather underworked, seems inevitable, unless more money is obtained in order to pay their salaries.

### THE CORONER'S JURY AND THE NURSES AT WITHAM.

The jury who held an inquiry last week into the deaths of the persons killed in the disaster to the Cromer express at Witham appended to their verdict a wish to praise "the nurses and all others who assisted after the accident." We are glad that the nurses were not in this instance forgotten. No less than the three signalmen who prevented another catastrophe, and the medical men who attended so promptly upon the sufferers, the nurses merit the compliment which has been paid to them.

### LECTURES TO MIDWIVES.

The lectures which were given to practising midwives in seven centres during the earlier months of the year, under the auspices of the London County Council, will be continued in the autumn, and we understand that further classes will be formed all over London if a sufficient number of midwives signify their desire to attend. These lectures are practical, and are delivered by medical men. District monthly nurses will be admitted to them, and the fee for the course of thirteen lectures is only one shilling. Any midwife expressing a wish to attend to the Secretary for Promoting the Training and Supply of Midwives, Dacre House, Dean Farrar Street, Westminster, S.W., will be notified of the nearest centre where the lecture will be delivered.

### SALARIES IN AUSTRALASIA.

We observe that the authorities of the Wangaratta District Hospital, Australia, advertise for a matron at a salary of £70. She must be able to impart instruction and practical demonstration to pupil nurses, be proficient in economic hospital administration and aseptic surgical work; and a member of the Victorian Trained Nurses' Association. A certificated nurse for the same hospital is also advertised for at a salary of £40 per annum. She is required to do alternate night and day duty.

### DISTRICT NURSING AT GLENGARIFF.

Continuing her visits of inspection in Ireland this month the Countess of Dudley, during her stay at Killarney House, spent some time with Nurse Walshe, who is working at Glengariff. Before she left the district her Excellency made a point of offering her thanks to the priest and the people for their great kindness to the nurse. She also inspected the site for a cottage which is to be built in Castletown Road as a permanent residence for the nurse. The personal interest evinced by Lady Dudley in the progress of the nurse, whose appointment is due to her energy, cannot fail to augment the success of the movement.

### PROGRESS IN THE ISLE OF WIGHT.

The committee of the Newport and District Nursing Society in the Isle of Wight are to be congratulated upon the contents of the sixteenth annual report, which was discussed at the meeting of subscribers the other day, and also on the balance-sheet. The former shows that the duties of the two nurses were admirably discharged, and that the services of the maternity nurse for the first year were greatly appreciated by the poor. As to the financial position, the receipts amounted to £208, as against expenditure £171, and in addition to this substantial credit balance there is a guarantee fund for the maternity nurse of £50 at the bank. We are glad to learn the Queen's nurse who paid 4,689 visits, was relieved in order to obtain an adequate holiday, her substitute paying 338 visits.

### A BEGINNING AT SEGHILL.

Under influential local auspices a nursing association has been started at Seghill in Northumberland. At the meeting called to consider the question an address was delivered by Miss White, superintendent of the Northumberland Nursing Association at Morpeth. It was decided to organise house-to-house collections at once. This is an admirable plan, and should invariably be followed in the initial stages of forming an association. It is the best mode of advertisement, and the most likely to ensure a representative subscription list.

### SHORT ITEMS.

The marriage took place at St. Barnabas, Middlesbrough, on Monday of Miss Bessie Evans, nurse at the Sanatorium, Middlesbrough, to Mr. R. Harbutt, of Wallsend-on-Tyne. The numerous presents included a silver sugar-bowl from the medical officer, silver sugar-tongs from the matron, and a drawing-room clock from the nurses of the Sanatorium.—On Thursday last week, Miss Terrill, night superintendent of the General Hospital, Tunbridge Wells, was married at the Wesleyan Church, Exmouth, to Mr. Herbert Drummond, of Southampton. The bridesmaids were Nurse Ethel Terrill, of Portsmouth Hospital, and Sister Nora Evans, of Shrewsbury; and among the presents were several from the matron and staff of Tunbridge Wells Hospital.

### The Nursing Outlook.

"From magnanimity, all fear above;
From nobler recompense, above applause,
Which owes to man's short outlook all its charm."

### NURSING IN AUSTRALASIA AND NEW ZEALAND.

In New Zealand the hospitals and training schools are under the control of the Government, and in Australasia the Government subsidies are so large that each institution and school is subject to Government inspection, and so the enforcement of a system of nurse registration was a simple matter. In circumstances like these, when the Legislature passes an Act, its enforcement becomes practically a matter of Government routine. In the United Kingdom, under the voluntary system, no Act which Parliament is ever likely to pass can have any such results, unless it is backed by the authorities of the chief Hospitals and schools. Here lies the crux of the difficulty so far as the old country is concerned, and a knowledge of this fact enforces the necessity to secure the co-operation and the inter-relation of the nurse-training schools and of all hospitals which receive probationers for training.

We propose to deal first with Australasia, leaving New Zealand to be treated in a subsequent article. Australasia has two nurses' associations, one the Australasian Trained Nurses' Association with head-quarters at Sydney, which includes all the States of the Commonwealth except Victoria. In Victoria there is the Royal Victorian Trained Nurses' Association, which was founded very soon after the Australasian one, and on very similar lines. These two associations have hitherto had most cordial relations, but a difficulty has recently arisen, out of an endeavour on the part of the Australasian Council to remedy an anomaly resulting from that association having, under the reciprocal agreement, to admit all members of the Victorian Association who may apply for membership. This regulation did not affect the nurses trained at the larger hospitals in Victoria, where the conditions of training are very similar. "In the smaller hospitals in the States, where the Australasian Association is established, the period of training is extended when the daily occupied beds sink below a certain number; in Victoria the same period of three years is considered sufficient for all cases." It can readily be surmised that three years' residence in a very small hospital, where few cases are received, and those usually of a relatively trivial nature, cannot possibly afford sufficient facilities and material for the adequate training of a probationer. Hence the difficulty the Australasian Association has experienced in regard to admitting all members of the Victorian Association. This objection has caused the Victorian Association to question certain features in the training in vogue under the rules of the Australasian Association. These objections include "the absence of central examinations, the registration of midwives without a certificate of general training, and the recognition of private hospitals as training schools." These questions are still under discussion, and every possible endeavour is being made to restore the former satisfactory inter-relations between the two associations.

Another point of interest is the history of the benevolent fund of the Australasian Association, which has been in existence for more than five years. It aims at providing funds and assistance when illness overtakes a nurse, and she is so temporarily or permanently disabled. The fund was instituted to prevent nurses in such circumstances from becoming dependent entirely on the charity of their friends, upon a system of self-help, whereby each member of the association is invited to subscribe half a guinea per annum to the benevolent fund. At the end of five years the funds do not amount to £300, whilst the voluntary subscriptions last year yielded about £15, or some 3d. per member. One reason for the relative non-success of this fund appears to be the doubt which exists in the minds of the council of some of the branch associations in different States as to whether it is better to join the central fund or to form a local State fund on similar lines. Apparently no local State funds have as yet been established, and it is very desirable that some steps should be taken to deal with this important matter on its merits, and to come to a definite conclusion so far as the members in each State and the whole of the members of the Australasian Association are concerned. In our view it would seem to be desirable that the officers of the association should place themselves in communication with the secretary of the Royal National Pension Fund for Nurses, 28 Finsbury Pavement, London, E.C. This fund was established to make provision for the needs and security of every working nurse throughout the British Empire, and there can be little doubt that a scheme of affiliation might readily be entered into between the Australasian Association and the Fund, whereby the more thrifty amongst its members might readily provide for themselves in the safest and most advantageous manner at a cost well within their means. We hope shortly to deal with other points in connection with nursing affairs in Australasia, which cannot fail to interest English nurses in whatever part of the Empire their work may be carried on. The whole number of nurses there is relatively small (under 3,000 we believe), but they make up in energy and public spirit for any disadvantage which might otherwise attach to them from this fact. May they go forward and prosper increasingly.

### Medical Electricity and Light Treatment.

By Kate Neale, Sister-in-Charge of the Actino-Therapeutic Department, Guy's Hospital.

### VIII.—*X*-RAYS.

It was not until some little time after the *x*-rays were discovered by Röntgen in 1896 that they were found to possess a value in medicine quite apart from their use as a means of revealing fractures in bones. Gradually, however, it became recognised that a patient who was often exposed to their influence was liable to develop a peculiar and chronic inflammation of the skin (dermatitis) and that this untoward result was also apt to appear in those whose work brought them in frequent contact with the rays. Once this effect was appreciated it was but a natural surmise that if the rays could irritate the healthy skin they might, perhaps, favourably influence the course of chronic skin diseases. The test of practical experiment proved the hope to be well founded and the rays have taken a prominent place in medical treatment.

With *x*-ray photography we are not concerned here, for it is a subject that lies beyond the confines of a nurse's duties. I shall restrict this chapter to an account of the application of the rays in the treatment of disease as far as concerns a nurse.

### What are X-rays.

*X*-rays are generated by passing the current from an induction coil through a glass tube from which almost all the air has been removed. Such a tube is known as a **Crookes tube**, from its discoverer, Sir William Crookes, and one form of it is represented in fig. 13. You will see that it consists of a large globe out of which lead two or more often three short glass tubes. A is known as the **kathode**, B, facing the kathode, is the **anti-kathode**, and C, the **anode**; B and C are joined by a wire. Each of these terminals ends inside the tube in a metal plate or mirror, that of the kathode being cup-shaped and of platinum.

To understand how the rays are generated you must remember that although the tube contains almost a vacuum there remains in it a trace of air, the atoms of which are capable of receiving a charge of electricity, and if the induction coil be connected to the anode and kathode of the tube the atoms near the

Fig. 13.—Crookes Tube.

latter become charged and are then repelled from its metal cup. Since the tube contains comparatively few atoms to get in each other's way those charged with electricity can rush away from the kathode with enormous velocity. Before they have travelled far, however, they strike against the anti-kathode mirror, and by the force of their impact give rise to the *x*-rays. These latter are then reflected from the anti-kathode mirror out of the tube. The object of making the kathode mirror concave or cup-shaped is to focus the atoms on the centre of the anti-kathode mirror, and in this way cause the reflected *x*-rays to be concentrated in one strong beam. As a matter of practice it is found that if the anti-kathode mirror is exactly at the focus of the kathode mirror, so great is the force of impact of the atoms, that its platinum will be melted. To avoid this, care is taken in the manufacture that the anti-kathode mirror shall be just a little out of focus.

A Crookes tube does not last indefinitely because by frequent use the small amount of air it contains grows even less, with the result that it offers an increasing resistance to the passage of a current. An old tube that has lost much of its air is spoken of as one of high resistance, or more shortly as a "high" or "**hard**" tube, while a new one, with a low vacuum, is said to be "low," or "**soft.**" This difference has a practical bearing in treatment. It is customary to measure the current of the induction-coil by a milliampèremeter, so as to know that *x*-rays of the proper strength are reaching the patient. Now, you may find that one tube will run easily with a current of, say, two-tenths of a milliampère, yet the next tube you use gives an unsatisfactory result with exactly the same current, and requires three- or four-tenths of a milliampère for its proper working. The second tube, then, will be a hard tube, and you will at once see that with each change of tube you must readjust the strength of the current.

*X*-Rays have the power of passing through the soft tissues of the body, but they are unable to traverse lead or any substance containing lead. We shall see later how this property that lead possesses is made use of in treatment to protect the healthy skin of the patient from the harmful action of the rays.

### Apparatus Used in Treatment.

The induction-coil is worked from a switch-board provided with both collecting-dial and reverser. The two wires from the coil are joined to the anode (or to the anti-kathode) and to the kathode of the tube, but if a milliampèremeter is being used, one of the wires must pass to a terminal of this, the other terminal being joined to the tube. The latter is supported in position by a shield and stand. The shield (fig. 14) is a hemispherical globe of lead-glass, out of the centre of which a window is cut. The tube is fastened inside the shield and the window directed towards the patient. None of the rays can pass through the lead-glass, and therefore the sole place at which they emerge is the window, and thus the shield protects the patient from injury. When the diseased area is small even this window would allow too extensive a beam of rays to pass, and in these cases you must fix into the shield one of the glass focussing tubes shown in fig. 15. These are of varying shapes, open at both ends, and the expanded part is fixed by a catch to the window of the shield, while the smaller end touches the patient's skin. By this means the area exposed to the rays can be nicely graduated.

The shield and focussing tube can be dispensed

Fig. 14.—*x*-Ray Shield-Stand.

with if a Cossar's vacuum tube be used (fig. 16). This is a form of Crookes tube, but is made of lead-glass and has a large side-tube, down which the rays pass. The end of this side-tube is of ordinary glass, and forms the only site at which

Fig. 15.—Focussing Tubes.

the rays can escape. Incidentally I may say that lead-glass can be recognised by its bluish tint.

When a tube is used without a shield or focussing-glass—as, for instance, in applications to deep-lying organs, like the spleen—the distance from the anti-kathode mirror to the skin over the organ should be about eight inches. Remember that the rays are just as likely to injure your skin as the patient's, and you must take special care not to expose yourself to their action. If it is necessary to handle a tube for any length of time you will be wise to protect your hands by gloves lined with lead foil. They are easily made after the pattern of a child's glove, of velvet, stitched and bound round.

### How to Treat.

Place the Crookes tube in position in the shield, so that the anti-kathode mirror is directed towards the window. If this precaution be omitted treatment will be useless. Fit into the window a focussing tube with a diameter just sufficient to cover the

Fig. 16.—Coosar Tubes.

area you are going to treat, and connect the induction coil to the *x*-ray tube and milliampèremeter in the manner described above. Put the patient in position so that the affected area of skin is pressed gently against the end of the focussing tube. You should take a little trouble to make him comfortable in whatever position he is to remain, else he is apt to grow more or less cramped before the treatment is finished. Lastly, switch on the current. If this be done gradually there is less chance of injuring the tube.

The doctor will always have given instruction touching the strength of current to be used, and you must therefore keep a frequent eye on the milliampèremeter to note any alteration in the reading. You regulate the current by means of the collecting-dial on the switch board. The milliampèremeter in common use is graduated in tenths of a milliampère, and the usual strength employed is from one to two-tenths of a milliampère. If the current runs as high as three- or four-tenths, the anti-kathode mirror is liable to grow red-hot.

The patient must not be left during treatment, and you must carefully watch that the right strength of current is maintained. After treatment has been applied to an ulcerated area (*e.g.* rodent ulcer) a dressing must be applied, but if the skin is unbroken or the treatment has been given to a deeply-situated organ such as the spleen, no covering is required. Any part of the apparatus that has come in contact with the patient must be thoroughly carbolised (carbolic lotion, 1–40) and dried before further use.

In cases where a Crookes tube of ordinary glass is used without either shield or focussing tube it is essential to protect the patient's skin from burning by expansive sheets of lead-foil. They must be

### MEDICAL ELECTRICITY AND LIGHT TREATMENT—*Continued.*

arranged right up to the edge of the area under treatment, and the same precaution must be taken if necessary to insure the safety of the eyes. After use this foil, too, must be carbolised.

Treatment extends from five to fifteen minutes, and is repeated at varying intervals according to the disease. Thus with rodent ulcer the treatments are often given daily, while with ringworm much longer intervals are necessary. If the rays produce any local inflammation, as shown by redness or swelling, no second treatment must be applied to the area until this reaction has subsided.

It will sometimes happen that the tube begins to work badly and the light from it becomes intermittent and flickering. As soon as you notice this, reverse the current at the switch-board for a moment and then turn on the collecting-dial for two or three knobs. Another difficulty arises when a specially "hard" tube is employed. The current is unable to overcome its resistance even with the collecting dial switched nearly full on. In such a case try the effect of heating the tube. Either place it in front of the fire for a few minutes or carefully warm it by a spirit lamp.

### Dangers.

Without doubt the chief and most serious danger associated with *x*-rays is the occurrence of dermatitis. This may occur in either patient or operator, and appears any time after treatment up to about three weeks. At first a mere redness of the skin, the affection may pass on gradually to ulceration, and a year may elapse before the sore is healed. In some cases the only remedy lies in amputation, and instances are known in which the ulcerated part has developed cancer. You will therefore appreciate how very serious is the danger that may follow carelessness in the application of the treatment.

### Diseases Treated.

The rays have now been used in many different diseases, and there are some few conditions which are peculiarly susceptible to their action. Of these rodent ulcers are perhaps the best instance, though lupus is also benefited to a large degree, and many satisfactory cures of ringworm of the scalp have been effected. Secondary malignant growths are not infrequently treated by the rays, as also are certain other conditions such as the enlarged spleen of leucocythæmia.

### The Nurses' Clinic.

### THE DISPENSARY. BY A CERTIFICATED DISPENSER.

### MEDICAL BOOK-KEEPING.

Many dispensers are called upon at some time in their career to keep doctors' accounts, so I propose in this article to try and give an idea as to how it may be satisfactorily done, for if a novice attempts to keep books without any previous knowledge of the subject the result will be a glorious confusion and at the end of the quarter or year, as the case may be, neither the doctor nor the book-keeper will be any the wiser as to whether the balance be on the debit or on the credit side! Four books will be required, namely, a ledger, a day-book, a bill-book, and a little one for entering the small sums received in the surgery. There are three classes of patients for the scale of charges; one for those whose house rent is from £10 to £25 per annum, the second for those who pay from £25 to £50, and the third whose rent is from £50 and upwards, and attendance on servants is made at a reduction in proportion. A night visit is charged for as double an ordinary visit; and then there are special charges for midwifery, for administering chloroform, gas, etc., and for consultation with another practitioner. There are certain Latin abbreviations which the book-keeper will need to know by which to indicate the nature of the case attended to and the charge which should be made, of which the following are the principal ones:—

Att. (attentio) signifies called; ita, a visit; att. chir. (chirurgical) surgical attendance; surg. noct. or surgiens nocte, rising at night. C.O. cura obstetrica, obstetrical case; C.O. puer., signifies a male, and C.O. puelle, a female; or it may be put, Partus pueri, partus puellæ; Ext. Dent., dentis, extracting teeth; Aperiens Abscessus, opening an abscess; Incisis Gingiveris, lancing gums.

In the ledger one end of the book must be kept as a "Where is it?" for patients' addresses, and after their name and address the number of the pages on which their accounts are entered. Things that are paid for at the time are not entered in the ledger, only in the day-book, and in the small book kept for cash received in the surgery. In the ledger the accounts are put down in this manner:—

Simpson, Mrs., 1 Charleville Street.

March 4th: Ita, 2s. 6d. 6th: Ita, 2s. 6d.; M., 2s. 10th, M., 1s. 6d.

Brown, Col., 14 Park Street.

March 11th: Ita, 10s. 6d.; M., 3s. 6d. 12th: Att., 5s. 14th: M., 3s. 6d.

M. signifies medicine. A certain number of lines are allotted to each person, and when the bills are sent out the lines are added up and the amount put on the right hand side. It will be noticed that the word Ita or M. is placed on the line, while the amount charged is put in small figures above it.

In the day-book the entries are made as follows, first the name of the day, in Latin always, as:—

Die Mercurii (Wednesday) July 20th, 1902.

Smith, Mr., 14 Cromwell Street.
Ita, 5s.

Then follows the prescription Mr. Smith has made up, and the price of it on the right-hand side below the 5s. for the visit.

Robinson, Miss, 72 East Street.

Att, 2s. 6d.; pills, 1s.

When the bill is paid the amount is put on the left-hand side and initialed by the receiver and the whole thing crossed off with a straight line from top to bottom. When, however, it is not to be charged for, the amount which should have been paid is recorded on the right-hand side of the line, a little cross or mark is put in the left-hand margin and the whole crossed off with a diagonal line. At the end of the day the names and unpaid accounts, but not the prescriptions, are entered in the ledger as shown above. It is better to have a sort of "Where is it?" at the end of each book, or a book kept entirely for the addresses with reference to the pages in each book in which the addressees' particulars are to be found. In writing prescriptions in the day-book great care must be taken to copy them accurately and distinctly, and each must be numbered, and if a word is illegible it must not be guessed at but returned to the writer to rewrite it more plainly. In the Grand Duchy of Hesse Darmstadt the Government has lately decided to hold the dispenser responsible if there is a fatal result owing to an illegible prescription, for not having sent the prescription back and insisted on having it distinctly written. For reference or repetition the folio number in the day or prescription book can be put on to the label of the bottle, or simply the date, or it can be done by a code of symbols or letters. Stock mixtures are best either numbered or distinguished by a letter, so when the dispenser receives a bottle back for a "repeat" it will be known at once what is required by the number or letter on the label without having to look in the book first. It saves a good deal of writing by simply putting in the day-book Mrs. Smith, Ita, 2s. 6d., 47 1s., which would mean that she had the stock medicine numbered 47, instead of writing out at length the whole name or prescription.

The drugs, too, can be distinguished by letter, and thus save time when the prescription has to be written as B for bismuth, O for tinct. opii, etc.

To ascertain at a glance who the patient is who brings the bottle, whether belonging to the club or to the parish, or is a private individual, the prescription number or abbreviation can be placed on the left hand side of the name in the case of a club patient, on the right side for a private person, and on top for a parish case; thus:—

48
Miss Amy Desmond
57 Mr. William Brown
Mrs. Johnson. O m x B gr. xx Aq$CHCl_3$.

indicates at a glance that Miss Amy Desmond comes from the parish, and requires the stock medicine No. 48, that Mrs. Johnson is a private lady who is ordered ten minims of tinct. opii and 20 grains of bismuth to each dose, and the bottle filled up with chloroform water—$CHCl_3$, being the formula for chloroform; and that Mr. William Brown is a club patient.

Of course there are many other methods by which bottles can be distinguished, and very many patients seldom return their bottles at all, thus obviating all difficulty in finding out what was in them. If possible, parish and club patients should be made to return them, or pay a penny or a halfpenny on each, as the doctor's expenses may be materially increased by having to provide each patient with a new bottle every time.

In the bill-book, as its name indicates, on the left page all the bills are entered when they are sent out:—

| | |
|---|---|
| Brown, Col., 14 Park Street ... ... | £5 5 0 |
| Simpson, Mrs., 1 Charleville Street ... | £7 10 0 |

and so on; and on the right-hand page are entered the bills paid. At the end of the bill-book is a summary of payments and expenses, by reading down which anybody can see at a glance exactly in what state his affairs are. On the left side of the page are placed the receipts—

| | |
|---|---|
| Bills paid ... ... ... ... ... ... | £400 |
| Cash in Surgery ... ... ... ... ... | £50 |
| Appointments ... ... ... ... ... | £150 |
| | £600 |

while on the opposite page are put the expenses—

| | |
|---|---|
| Drugs ... ... ... ... ... ... ... | £50 |
| Dispenser's salary ... ... ... ... | £75 |

and the many other expenses that may occur. If the accounts are put down accurately and carefully every day, and checked either monthly or quarterly, the book-keeper will have very little trouble with them and the doctor will have the satisfaction of knowing exactly how he stands. The accounts can always be proved by comparing the different books with one another.

### Incidents in a Nurse's Life.

**Contributions for this column are invited.**

### A COTTAGE HOSPITAL EXPERIENCE.

OWING to the smallness of the staff the experiences of a matron or nurse in a cottage hospital are very varied, and one's powers of endurance are sometimes heavily taxed, as in the following case.

An elderly man was admitted to our little hospital suffering from a popliteal aneurism. The doctor, a young and up-to-date surgeon, decided to attempt a cure by digital pressure. He himself was too busy to give much actual assistance, but he inspired us with his enthusiasm, and also promised to place his dispenser at our service to take his turn in applying pressure.

We started the tedious operation one day about noon. The doctor, with a 2-lb. weight wrapped in cotton-wool, applied pressure to the femoral artery until all pulsation ceased in the aneurism. The dispenser then took his place, and the doctor, after seeing that I understood his instructions, departed, leaving us to our difficult task.

We found the dispenser utterly useless. Either from laziness or from incapacity, he did not exert sufficient pressure to stop pulsation in the artery. I therefore thanked him for his help, and suggested that perhaps we could manage without him, as it must be his dinner hour. With great alacrity he made off, and then the tug of war commenced.

Almost the whole work fell upon one nurse and myself. One of us kept up pressure, whilst the other sat with her hand over the aneurism in order to detect the slightest pulsation. Every ten minutes we cautiously exchanged positions, until we wondered whether we could possibly hold out longer, and the minutes dragged out like hours.

The patient was brave, but he felt terribly bad for a time, and it required all our powers of persuasion to induce him to persevere.

During the afternoon the doctor looked in to encourage us; the district nurse gave about an hour's help, and another doctor on the staff also gave us thirty minutes' assistance. In spite of these little breaks the hours rolled slowly on until about 7.30 P.M., when the doctor came again and gave orders for our work to cease.

We forgot our cramped and aching limbs in our anxiety to hear his verdict as he examined the result of our labours. To our joy he declared that there was no pulsation, and he hoped for a good result.

The next morning, however, a slight beating was to be felt, and we returned to our work for about a couple of hours. From that time all pulsation finally ceased, and in due course the old man was about again and cured.

The case found its way into the medical journals, but I thought in reading the concise account from what different standpoints one can view the same facts! To the ordinary reader it was just an account of an interesting case, but to us it brought a vivid picture of hours of strain and tension whilst a human life was being fought for and the victory won.

### To Nurses.

WE invite contributions from any of our readers, and shall be glad to pay for "Notes on News from the Nursing World," or for articles describing nursing experiences at home or abroad dealing with any nursing question from an original point of view, according to length. The minimum payment is 5s. Contributions on topical subjects are specially welcome. Notices of appointments, letters, entertainments, presentations, and deaths are not paid for, but we are always glad to receive them. All rejected manuscripts are returned in due course, and all payments for manuscripts used are made as early as possible after the beginning of each quarter.

### The Hospital Salpêtrière, Paris.

BY AN OCCASIONAL CORRESPONDENT.

Upon entering the porch of the great Salpêtrière Hospital, Paris, a stranger is bewildered at the vastness of the place; one sees a long perspective of archways to be traversed, with directions over each indicating the different sections into which the hospital is divided. Not having been wisely directed, I wasted an hour waiting to see one of the staff, who, after all, could not give me permission to visit the wards. Then I was advised to go to the director's office, where I discovered an official with whom I had a long conversation about nursing in England and the prospects of hospital nursing in Paris. He afterwards sent for a nurse and gave her instructions to show me all the most important parts of the building, especially the surgical wards. The hospital, which in the time of Louis XIV. was used as a prison, contains beds for 5,000 patients, and admits every kind of case except maternity. My guide led me first to a small pavilion for women devoted to serious operation cases. The wards were light and airy, painted walls and tiled floors, bedsteads all painted white, and white coverlets to the beds. A special operation-room with all the newest appliances for aseptic surgery is one of the features of this pavilion. The largest ward contained six beds; a relieving touch to the whiteness of this ward was a beautiful castor-oil plant, which stood upon a small table. The other wards were all small, but looked cosy and attractive.

### The Surgical Ward for Women.

Our next visit was to the surgical ward for women, which is a large, lofty, and imposing ward. It has rather a peculiar appearance from the presence of two rows of solid pillars like one sees in a church, these are evidently a relic of the old prison days. The walls are painted a delicate green, and the ceiling white. The bedsteads are enamelled white, and small white lockers stand beside each bed. These lockers have no doors, and it seemed a slight drawback that such a number of articles remained all day upon the locker-tops, bottles of wine, fruit, etc. I saw only one temperature chart; on the Continent it is not the fashion to leave either treatment cards or charts within reach of the patient. This plan has its advantages on visiting days for obvious reasons. Another relic of former days was a handsome antique chest with brass fittings. The nurses seemed very proud of it, but it appeared to me a curious appendage to a surgical ward. The tiled floor, I was informed, is washed over every day, quite an undertaking it must be. In this ward was a white-painted screen, with white draperies, of this fact I made a special mental note, as the lack of bedside screens in Continental hospitals had often struck me painfully on former occasions. The surveillante or head-nurse of this ward told me she had been there for ten years, she looked very bright and capable with her flashing eyes and red-gold hair. She showed me everything with great pride, her operation-room with all its sterilising apparatus, glass barrels for antiseptic lotions, and hot-air cupboard for linen cloths and doctors' jackets. The light enamelled operation-table, in three movable pieces, so easily cleansed and disinfected, was a model of efficiency. In addition to her large ward, in which every patient was an operation case, this nurse had charge of a smaller ward for old and infirm women, and still another for two or three old men. She had two assistants and a first year probationer who did all the cleaning and attended to the linen; at the end of the large ward was a bath-room and lavatory and small kitchen for the heating of water in great copper boilers. I was quite convinced those nurses worked very hard, and yet they looked quite cheerful and contented.

### The Lunatic Wards.

We then, having seen everything of interest in the surgical ward, proceeded to the lunatic wards. These are arranged on the pavilion system. Here it was cleaning-day and the patients were helping to polish the floors; further on a number of women were contentedly sewing, all looked tidy and well-cared for. The nurse who accompanied me assured me that they could be easily managed by kindness, and it was not difficult to see that she was on excellent terms with them all. The dormitories, containing about 20 beds each, were orderly and well-ventilated; a very few patients were in bed. A large square garden provided space for exercise for the 200 female patients. From the lunatic wards we went to the museum, where I was shown some very uncanny-looking objects; models of abnormal limbs in glass cases, a full-length figure representing extraordinary deformity, and other interesting but more or less dreadful objects. After the museum came the electric department, where nervous diseases are treated. The head nurse explained rapidly the use of the various appliances, but being Saturday and also cleaning day, I could not see everything to advantage. The children's ward came next. This was a long bungalow ward; most of the patients had gone to school, I presume within the precincts of the hospital grounds, only a few remaining in bed. One paralysed girl was busy making doll's clothes, she seemed contented in spite of her sad affliction. I was sorry not to see all the other occupants of the 25 beds, yet it was well for them that they were able to get up and go to school.

### The Kitchens.

Lastly I saw the kitchens and they were a sight to be remembered. A long building, as large as many of our largest wards, was in the hands of white-jacketed cooks, who must have a busy life of it. After cooking the mid-day meal for their 5,000 clients they were enjoying a slight respite from their labours. A regular army of copper boilers were shining like burnished gold, and kitchen utensils hung upon the walls in imposing array. At one end of this immense kitchen was a department that looked like a butcher's shop. Here were displayed legs and shoulders of mutton, sides and haunches of beef, and heads and tails of both. All was in excellent order, and reflected great credit on the organising qualities of the culinary manager-in-chief, though I did not happen to see him. About midday women were hurrying in all directions with appetising-looking dinners. The first meal of the day is at 7 a.m., the next at 11.30, and the last at 4.30. This rule is for ordinary diet; different regulations, of course, are carried out for fever diet and special cases.

### Nurses and their Training.

The nurses' uniform consists of holland dresses, white turn-down collars with a small black ribbon bow to fasten them, white aprons, and small stiff caps; these caps vary according to the grade of the nurse. The head nurse wears a black one, the next in rank wears a white one with a black stripe on the loops of the front bow, and the third and fourth nurses wear a white one, pure and simple, with the stiff little bow in the front and two stiff streamers hanging down behind. The fourth nurse wears a blue dress, and is equivalent to our wardmaid in England, except that she has the privilege of rising to be a surveillante if she is sufficiently capable. The nurses are received first for a month on trial, then they go through a course of training lasting from October to July; this includes lectures on physiology, anatomy, nervous diseases, etc., and bedside lectures on the various cases in the wards. After this course of training they are tested by a severe examination, and may then be drafted off to another large Paris hospital. No nurse can become a surveillante until she has passed her examination and received a diploma. The pay of the nurses begins at about 28s. a month, and may rise to £48 a year. They go on duty at six in the morning and leave the wards at 6 P.M., when the night nurses arrive. They have a half day off once a week, and ten days' holiday once a year. Every nurse's linen is marked with her own number, so that she runs no risk of using other people's. I saw a new building in course of erection which will provide bedrooms for 100 nurses. After spending three hours at the hospital, I began to feel tired and hungry, though still greatly interested in all I had seen. Just before leaving my guide took me through the church, which, like all Roman Catholic churches, looked rather cold, though very clean and well-kept. I noticed that many of the nurses were engaged in office work; it seemed very droll to hear them call each other "Mademoiselle" or "Madame," but there seems no exact equivalent to our title of "Nurse." They seemed very pleased to welcome an English nurse, and I was equally charmed to see French nurses at work, and bade them farewell with sincere regret.

### Nurses on Holiday.

A TRIP TO BRUGES. BY A PRIVATE NURSE.

"Where shall I go for my holiday? It has been a bad year and I cannot afford an expensive one." How often we have heard these words during these last few weeks, and to those whom it may interest I am going to relate the experiences of my own holiday, costing under £4 all told. To start with, we left early one lovely morning from St. Katherine's Wharf, Tower Bridge, by the Steam Navigation Company's boat *Alouette*, and had a long, lovely day on the sea, reaching Ostend about 7.30 in the evening. All the way there is something of interest. As you pass down the river there are some of the most picturesque landmarks of our riverside—Woolwich and Greenwich. For some time all sight of land is lost, and lunch on the boat occupies the attention of all those who were not yet wishing that the voyage was over. At last there is a sudden crowding to the fore part of the boat, and keen excitement prevails on seeing the coast of Ostend. There, on the quay, is the milkwoman, with her snow-white cap, and the milk cans slung from a wooden yoke over her shoulders, whilst her wooden sabots ring musically on the rough stones. Oh those stones! It seems impossible ever to get accustomed to them. How they make our feet ache! No wonder, indeed, that the peasants all wear their quaint old wooden sabots. At the quay there is an eager crowd of porters all waiting to seize your luggage; indeed, they do not wait until you are landed, but select their victim before the boat is at an anchor. Meantime, such a chatter rages; the porters for the luggage, the commissionaires from the hotels, each one sounding the praises of his particular house, that to those on board a wonderful mixture of French and indifferent English makes one wonder why there was ever a Tower of Babel. At last we are on shore, then quickly *en route* to Bruges, which place we reached at 9 o'clock, and made our way quickly to the convent, where the gentle nuns are ready to give tired nurses a rest within their hospitable walls for the moderate fee of 3.50 francs a day (or 3s. in English money). Everything is ready for us, a welcome supper of cold meat and fruit, then such a cosy room, looking over the dear old town of Bruges. On our right is the cathedral, stately, calm, and majestic; on the left the tall Belfry tower rises high above all else. From this tower, with its 40 bells, a lively little tune rings every 15 minutes, and the air is full of music. The nuns like us to be in early, and their doors are shut at 9.30; for their day begins at 5.30 in the morning. It is as well for the visitors, too, for sleep is impossible after six in the morning, those lovely chimes from the Belfry in harmony with many other bells in the town compel you to wake and admire them. Now for the town. Wherever you turn there is something fresh to see. To the picture lover there is all or more than she could desire in the costly collection of Memlings in the Hospital of St. Jean, the works of Van Oost, Van Dyck, and others, which abound in all the churches. To the artist there are all the picturesque bridges over the canal, the lovely old streets, with their artistic houses, the lacemakers at their cottage doors; everywhere these latter are to be seen, the old woman of 83 and the young child in her teens, sitting threading and switching their bobbins in and out with amazing rapidity. Some of the more elaborate patterns take 300 or 400 bobbins. They never make a mistake and never break a thread, it is simply wonderful to watch them, and we are thankful that all duty is now off Belgian lace.

Saturday is market day, and very busy and gay the city looks. Perhaps the part of the market most worth seeing is where the fish is sold by old women in white caps sitting under enormous blue umbrellas. Yet one cannot stay here long, as the odour is unpleasant, but pass quickly through to the pottery and copper stalls, which are very attractive.

Another day a trip up the canal into Holland costs us 2s., and we meet with the Dutch people, though it is sad to see how the Dutch costume is dying out on the outskirts of that country. Many may like to take a "season ticket" on the Belgian railway for five days, costing 9s. 3d., or for fifteen days, costing 18s. 4d. This carries you all over the country, to Brussels, Antwerp, and the Ardennes, and you can get a glance at some of the great cities and sights of Holland and Belgium, but it of course adds to the expense and the fatigue of the trip. To obtain this ticket you must have a small unmounted photograph of yourself, to be fixed on to your ticket for identification, and to prevent transfer of the ticket.

If you want to go to the sea the nearest place is Heyst, which you can reach for a few pence. The bathing there is perfect, and the long stretches of firm dry sand make capital tennis courts and cricket pitches, and you can have an exciting game, keeping an eye on the tide, which has a knack of creeping up unexpectedly and catching you or stealing your balls. At last, and all too quickly, our fortnight is up, and we must bid good-bye to the sweet nuns in their quiet and stately convent, and look out our return ticket for the boat from Ostend to London, which ticket only costs us 10s. 6d., and is available for two whole months. Our trip is over, and again the *Alouette* brings us back and lands us at St. Katherine's Wharf, all the better for a really good holiday, and ready for any amount of hard work.

### Death in Our Ranks.

We regret to record the death, on the 2nd inst., of Miss G. J. Lapworth, matron of "The Abode" (Nursing Home), Ryde, I.W., after a short illness. Miss Lapworth was trained at the Royal Infirmary, Sheffield, where she remained as sister for four years.

### The Royal Mail Steam Packet Company and their Nurses.

BY OUR COMMISSIONER.

"Particulars respecting the employment of nurse-stewardesses on the vessels of the Royal Mail Steam Packet Company can only be ascertained at Southampton," said the courteous manager of the stores department at the head offices in Moorgate Street where I called by appointment on Tuesday afternoon.

"But surely," I rejoined, with a little insistence, "as I have come here on a day to suit your convenience, it is possible for you to afford me some information. It is not sought out of idle curiosity, but for the benefit of the numerous nurses who send letters to the editor of The Hospital expressing their desire to know the conditions upon which they can enter the service of the company."

"We leave the arrangements entirely in the hands of the Southampton manager, though, of course, the policy of employing nurse-stewardesses is a matter which concerns the directors in London."

"How long have you been employing trained nurses on your liners?"

"About eighteen months. The number in the employment of the company is from ten to twelve."

"And the salaries offered—or is that one of the details which must be ascertained at Southampton?"

"I am afraid that I cannot enlighten you on that point. But I may say that we are inundated with applications for employment."

"From fully-trained hospital nurses?"

"Many of the applicants are, no doubt, trained nurses."

"How do you find the system work?"

"It depends upon the circumstances. Some hospital-trained nurses do not care about discharging duties which devolve upon the ordinary stewardess. The system works more satisfactorily, I believe, on vessels which carry more than one stewardess. On our new ship, the *Arragon*, we have three stewardesses—a fully-trained nurse as chief, a second nurse who is a probationer, and an ordinary stewardess."

"The Royal Mail Steam Packet Company was the first of the shipping companies to try the experiment?"

"Yes, and when a passenger is really ill, especially if an operation be required—as in the case which was mentioned in your columns—the presence of a trained nurse is very handy. I recollect an instance of an operation, however, which was successfully performed on one of our vessels, without a nurse."

"I suppose that on many voyages the services of a nurse are not required?"

"That applies to the great majority, and is the chief reason why we do not employ more. For sea-sickness without complications, the ordinary stewardess can do all that is essential."

"Then the duties of the nurse-stewardess are not very onerous?"

"Certainly not as a rule. If there are disadvantages in the shape of disagreeable work, the nurses probably find that their health is all the better for the sea-trip, and sometimes a grateful patient does not forget to recognise their services substantially. South Americans in particular are generous; I am rather surprised that nurse-stewardesses are not more in request on some of the big liners."

### Appointments.

Borough Fever Hospital, Dover.—Miss Margaret Woodley has been appointed charge nurse. She was trained at the Eastern Fever Hospital, London, and was afterwards senior nurse at the Southend-on-Sea Borough Sanatorium. She has since been engaged in private nursing.

Brighton and Hove Hospital for Women.—Miss S. L. Mumby has been appointed matron. She was trained at the London Hospital, where she was afterwards staff nurse and ward sister. She has taken matron's duty at Brixham Hospital, and the District Nursing Association, South Devon. She is registered under the Central Midwives Board, and also holds a certificate for massage.

Ebbw Vale Accident Hospital.—Miss Lily Alberta Houston has been appointed matron. She was trained at the Children's Hospital, Newcastle-on-Tyne. She has since been nurse at the Westminster Hospital, and ward sister at West Ham Infirmary.

Essex and Colchester Hospital.—Miss Emily S. Garside has been appointed matron. She was trained at Liverpool Royal Infirmary, and has since been sister at the Salop Infirmary, Shrewsbury, night sister at the Children's Infirmary, Liverpool, and home sister at the General Hospital, Birmingham.

Galashiels Cottage Hospital.—Miss Jessie A. Morison has been appointed matron. She was trained at Edinburgh Royal Infirmary, and has since been sister at the Kirkcaldy Cottage Hospital, and sister and dispenser at Arbroath Infirmary. She has also done private nursing at Stafford.

Infectious Diseases Hospital, Earlsheaton, near Dewsbury.—Miss M. Greenhalgh and Miss A. M. Bowden have been appointed staff nurses. They were both trained at the Bury Dispensary and Hospital, Bury, Lancashire.

Isolation Hospital, Swallownest, near Sheffield.—Mrs. Lavinia Holt has been appointed matron. She was trained at the Royal Infirmary, Newcastle-on-Tyne, and has since been matron at Eston Sanatorium, and Harrogate and Knaresborough Joint Hospital.

Monsall Hospital, near Manchester. — Miss Lucy Birkinshaw has been appointed sister. She was trained at Huddersfield General Infirmary, and has since been engaged in private nursing at Southampton and Bradford.

North Evington Poor-Law Infirmary, Leicester.—Miss Linda Kate Masters has been appointed assistant matron. Miss Bertha M. Bryant, Miss Jennie Duffy, Miss Ellen F. Jackson, Miss Sarah J. Bridgen, and Miss Martha E. Eldred have been appointed sisters. Miss Emily Hammond has been appointed midwifery nurse. Miss Masters was trained at Whitechapel Infirmary, where she has since been night superintendent. Miss Bryant and Miss Duffy were trained at Whitechapel Infirmary, Miss Jackson was trained at Leeds Infirmary, Miss Bridgen was trained at Shoreham Infirmary, Miss Eldred at Nottingham Infirmary, and Miss Hammond at Portsmouth Infirmary.

St. Neots Union Infirmary. — Miss Youngs has been appointed charge nurse. She was trained at St. Peter's Home for Sick Women, Kilburn, by the Meath Workhouse Nursing Association, and at Queen Charlotte's Hospital, London. She has since been nurse at Foleshill, Coventry.

### Presentations.

Southwark Infirmary.—Miss Massey, upon resigning her post as first assistant matron at Southwark Infirmary, East Dulwich Grove, London, has been presented by the medical, nursing, and domestic staffs with a handsome silver-fitted travelling-bag, a set of silver brushes and mirror, a silver-mounted purse, and a writing-case, as a token of their esteem.

### Everybody's Opinion.

### PORTERS AND ISOLATION HOSPITALS.

"G. M." writes: I have lately been appointed matron of an isolation hospital which, although only having a very limited number of patients at present, is capable of holding a great many. The hospital itself is in capital order so far as the nursing in the wards is concerned, but unfortunately the resident porter and his wife (who acts as laundress) are a sad trial. The man has been a lunatic attendant, and here he is supposed to clean the boots, knives and windows, do the disinfecting, answer the gate, and attend to the garden. He cleans the knives, objects to the windows, is indifferent to the gate, which is usually answered in consequence by one of the two maids, who have plenty of work to do elsewhere. As to the garden, in spite of extra help when required, the committee frequently have to buy fruit and vegetables from the town. The last matron, I hear, was asked to resign almost entirely because she failed "to get on" comfortably with this porter and his wife, who are paid respectively £52 and £32 per annum with house-rent, garden, etc. It is such people as these who are the bane of small county and isolation hospitals.

### A MATTER OF FORETHOUGHT.

"Assistant Nurse" writes from the Infirmary, Newton Abbot: With reference to your note regarding my application for leave, may I state the following facts? I had not thought of resigning until I saw the advertisement for a nurse elsewhere, therefore there was not time to have my holiday before sending in my resignation. I have been with the Newton Abbot Board of Guardians three years this week, during which period I have received two holidays; and I consider that to grant my request of leaving a week before the month on the condition that my salary was stopped the day I left most unjust and unfair. Our Board's rules are, after 12 months' work three weeks' holiday—salary not being deducted. Therefore, I think you will see I have not acted as you appear to infer I have done, for if I had known about this vacancy occurring, I should have had my holidays earlier. Under the present conditions I have most unfortunately had to go without. Still I trust that my health will keep good and not break down, as during my time here I have striven to do my duty to the best of my ability, and have acted about my resignation in a straightforward manner.

### DIET IN A SMALL HOSPITAL.

"A Busy Matron" writes: I am the busy matron of a small general hospital, but I find time to read The Hospital regularly, and, indeed, have done so for many years; this, however, is the first time I have ever penned a line in reference to anything I have read therein. I am, however, keenly interested in the subject of the daily menu for patients and staff. My hospital contains 26 beds, and the staff consists of two nurses, two probationers, and two servants. Our number of patients last year was over 300, and try how I may I cannot reduce the daily cost per head per day below 1s. 0½d. We live well but plainly; our patients are not allowanced, and I take the utmost care that there is no waste. When I read that the matron of an infectious hospital can feed her patients and staff on 6d. or so per day, I should very much like to know how it is done, at the same time taking into consideration that we are always allowed the best of provisions that can be obtained. I think the amount quoted by another matron—namely, 9s. to 9s. 6d. per week, is rather high. Of that, however, I will not be the judge. I should much like the opinion of other matrons, for it is, as I said before, a subject in which I am much interested. Another matter I should like an expression of opinion on, is the number of nurses and servants usually found in an institution of this size, so as to know whether this hospital would be considered under or over staffed.

[It is difficult to determine a fair average expenditure o provisions for "Busy Matron's" institution without knowing the daily average of occupied beds. She will find that the only way really to control expenditure is to disentangle the cost of the patients' board from that of the household. The household and staff should not cost more than a shilling a day a head, or if malt liquor is not provided rather less. The patients at an all-round estimate should not cost more than 9d. a day each, reckoning for the usual average (7 to 10 per cent.) on milk diet, and for about the same proportion of convalescents on extra diet, the remainder on ordinary diet. These are liberal estimates. Does the leakage take place in the *price* of provisions? "Busy Matron" should procure "Hospital Expenditure: The Commissariat" (Scientific Press), and study the effect of trifling variations in price on the average cost of provisions per head. The staff mentioned by "Busy Matron" is by no means excessive. The weak point is the attempt to train two probationers in a hospital containing only 26 beds.—The Author of the Article.]

### The Nurses' Bookshelf.

The Midwives' Roll. Printed and Published by Authority of the Central Midwives Board. (London: Spottiswoode and Co. Price 10s. 6d.).

The Roll contains a complete list of midwives registered as such up to March 31, 1905. The list comprises 22,300 names. The information within the volume includes the name and address of the midwives, the date of their enrolment, length of practice and any qualifications they may possess. The names of all members of the Central Midwives Board are given, also a list of institutions approved as training schools, and of approved teachers; a list of examiners, a scheme of examinations; the Midwives Act, 1902; rules framed by the Board; rules of procedure; and a table showing the totals of the various qualifications enrolled under Section 2 of the Midwives Act. The work is very complete, and is of a certain interest, but it is to be questioned whether the published list will be of much benefit or use to anybody. During the next few years, at any rate, the army of midwives is likely to largely change its *personnel*, and as a directory it cannot be of great value. This the editor indicates in a note at the commencement of the book in which he refers to the frequent changes of address which take place.

A Nursing Guide. 1905. (Issued by Guy's Hospital. Price 1s. 6d.)

We have received from the matron the third issue of this little guide, which is prepared yearly for the use of the nursing staff of Guy's Hospital. The present edition is somewhat larger than former ones, and a smoother paper has been used for its pages. In the text there are also alterations. There is a new chapter on district nursing, on medical electricity, and on the feeding of infants. The arrangements of the book are the same as before. It may interest both matrons and nurses at other hospitals to peruse the very full account of the nursing curriculum at Guy's Hospital, and all nurses may find something to learn in this very interesting volume. It contains advice and information for those about to enter the nursing profession, and chapters of practical points in such as sick cookery, massage, etc., besides those subjects we have already mentioned. Although the guide is in no sense a complete nursing text-book, it epitomises much that is useful, the matter being arranged very conveniently for easy reference. The Register of Nurses who have passed through the training school, and the movements of the staff within the hospital, the honours awarded, and the obituary, are of course of much interest to the nurses past and present of Guy's Hospital. The account of the Nurses' League, with its exceptional opportunities for recreation, may stimulate those at less favoured institutions to organise something of the kind for themselves.

### Notes and Queries.

**REGULATIONS.**

**The Editor is always willing to answer in this column, without any fee, all reasonable questions, as soon as possible.**

**But the following rules must be carefully observed.**

**1. Every communication must be accompanied by the name and address of the writer.**

**2. The question must always bear upon nursing, directly or indirectly.**

**If an answer is required by letter a fee of half-a-crown must be enclosed with the note containing the inquiry.**

*West Africa.*

(189) Will you kindly inform me about the West African Nursing Service?—*Miss R.*

Write to the Secretary, Colonial Nursing Association, Imperial Institute, London, S.W., who can give you information about nursing in most of the British colonies in West Africa.

*Midwifery Training Schools.*

(190) Will you kindly give me a list of approved midwifery training schools?—*Matron.*

This is contained in "How to Become a Certified Midwife" by Dr. Appel. The volume is published by the Scientific Press, 28 and 29 Southampton Street, Strand, W.C. (Price 1s. 6d.)

*Training.*

(191) I shall be much obliged if you will kindly tell me some particulars as to training for maternity work? Is there any hospital where women are trained, either for a small premium, or without one, so as to be fully qualified and secure certificate? Also kindly tell me of any hospital for general training on these terms.—*Miss H.*

See "How to Become a Nurse. The Nursing Profession: How and Where to Train," published by the Scientific Press, Limited, 28 Southampton Street, Strand, London, W.C. (Price 2s.)

*Former Training.*

(192) I have a certificate for two years' training at a hospital for women and children. Would you kindly tell me if there is any general hospital where they would take my former training into consideration?—*G. G. W.*

Write to the matron of Addenbrooke's Hospital, Cambridge.

*Dispensing Certificate.*

(193) Will you kindly tell me the cheapest and best way of gaining a dispensing certificate, after having gained one for midwifery and general nursing, and would it be possible to study for the same whilst following my profession?—*Anxious.*

Miss Buchanan, Gordon Hall, Gordon Square, W.C., has arranged special classes for dispensing for nurses, but you could not gain a public certificate without devoting a great deal of time to serious study.

*Massage.*

(194) Can you tell me of a good place where I can take lessons in massage?—*L. W.*

Write to the matron of the National Hospital for the Paralysed and Epileptic, Queen Square, Bloomsbury, London, W.C. Also to the Incorporated Society of Trained Masseuses, 12 Buckingham Street, Strand, London, W.C.; and, further, see our advertisement columns.

*Male Nurse.*

(195) I have a great desire to be a nurse but do not know how to proceed. Will you please tell me? I have passed the St. John's Ambulance examinations. My age is 22.—*F. W. H.*

Write to the matron of the National Hospital for the Paralysed and Epileptic, Queen Square, Bloomsbury, London, W.C.; also to the Army Medical Corps, 68 Victoria Street, London, S.W.

*Homes of Rest.*

(196) Could you tell me the names of homes of rest for nurses on the East Coast of England?—*Globule.*

Write to the lady superintendent of the Claughton Home, Walton-on-the-Naze.

**Handbooks for Nurses.**

| | Post Free. |
|---|---|
| "A Handbook for Nurses." (Dr. J. K. Watson.) ... | 5s. 4d. |
| "Nurses' Pronouncing Dictionary of Medical Terms." ... | 2s. 0d. |
| "Art of Massage." (Creighton Hale.) ... ... ... ... | 6s. 0d. |
| "Surgical Bandaging and Dressings." (Johnson Smith.) | 2s. 0d. |
| "Hints on Tropical Fevers." (Sister Pollard.) ... ... | 1s. 8d. |

**Of all booksellers or of The Scientific Press, Limited, 28 & 29 Southampton Street, Strand, London, W.C.**

### For Reading to the Sick.

"NOT AS I WILL."

"Not as I will:" the sound grows sweet
Each time our lips the word repeat.
"Not as I will:" the darkness feels
More safe than light, when this word steals
Like whispered voice to calm and bless,
All unrest and all loneliness.

"Not as I will," because the One
Who loved us first and best has gone
Before us on the road and still
For us must all this love fulfil
"Not as we will."

*Anon.*

"My God, my God, why hast Thou forsaken me?" These words are full of the deepest mystery, and they are also intensely practical, for they bring before us the experience of many a soul and teach us how to meet one of the great perplexities of human life—the experience of a sense of desolation of a sense of being forsaken by God. Sometimes it is in the sphere of spiritual experience that the sense of desolation is brought home to us. . . . Prayers become cold and apparently formal; meditation is most difficult; darkness seems to have rolled in upon the soul, darkness which hides God from our spiritual sight; and we cry, as our Lord did from the darkness around the Cross, "My God, my God, why hast Thou forsaken me?"

How we should thank our Lord for speaking these words which show us that He was indeed "in all points tempted like as we are, yet without sin"; that He in His human nature experienced all our sorrows and trials so that He can fully sympathise with us. But more than this, in these words, which our Lord quotes from the 22nd Psalm, He manifests the virtue of faith which is the fundamental virtue of the Christian life and "without which it is impossible to please God" (Hebrews xi. 6). Some may say, "Do these words manifest faith? Do they not rather manifest doubt?" No, they manifest faith. Had our Lord said, "O God, why hast Thou forsaken me?" There might be room for such a suggestion, but He said, "*My* God." He never for one instant faltered in His faith in God. He asked, as we may ask with reverence, "Why am I treated thus? Why do I suffer this sense of desolation?".

There are periods when God seems hidden from us; when, though we search into the darkness we can see nothing; though our ears are strained we cannot hear His voice. Then, perhaps, in our perplexity we may ask the question which our Lord asked, "My God, my God, why hast Thou forsaken me?" There must be in our spiritual life seasons of coldness, darkness, and desolation. There must be night and day, summer and winter, just as there are in our physical life. At such times we must exercise the virtue of faith and trust in God and falling back upon our remembrance of God's goodness to us in the past, find our answer in the promise, "I will never leave Thee nor forsake Thee." And say, like our Lord in His moment of supreme suffering, "Father, into Thy hands I commend My Spirit . . ."

*A. G. Mortimer, D.D.*

Sweet Saviour in mine hour of mortal anguish
When earth grows dim and round me falls the night,
O breathe Thy peace as flesh and spirit languish,
At that dread eventide
Let there be light.

*Hymns Ancient and Modern, No. 121.*

## Figures and Tables

**Fig. 13. f1:**
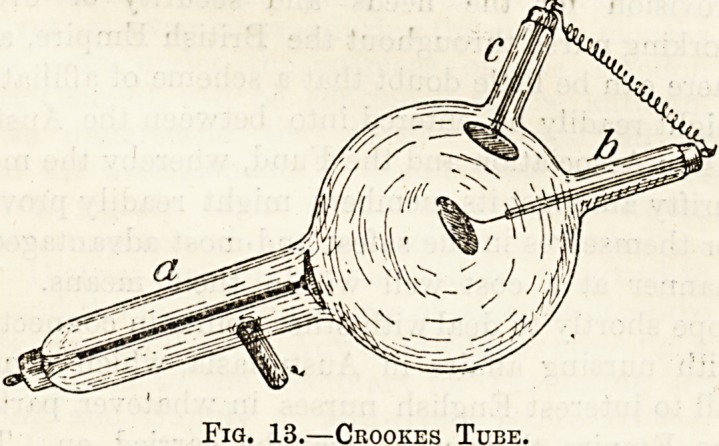


**Fig. 14. f2:**
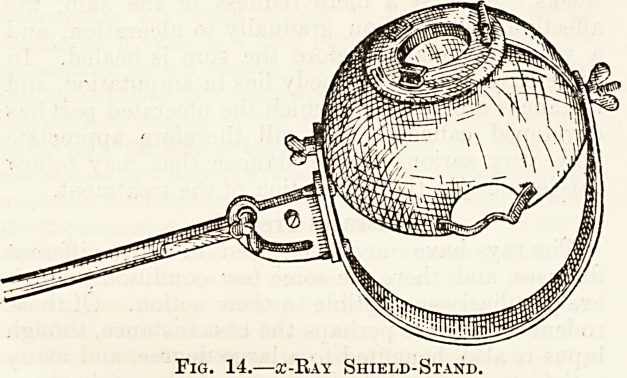


**Fig. 15. f3:**
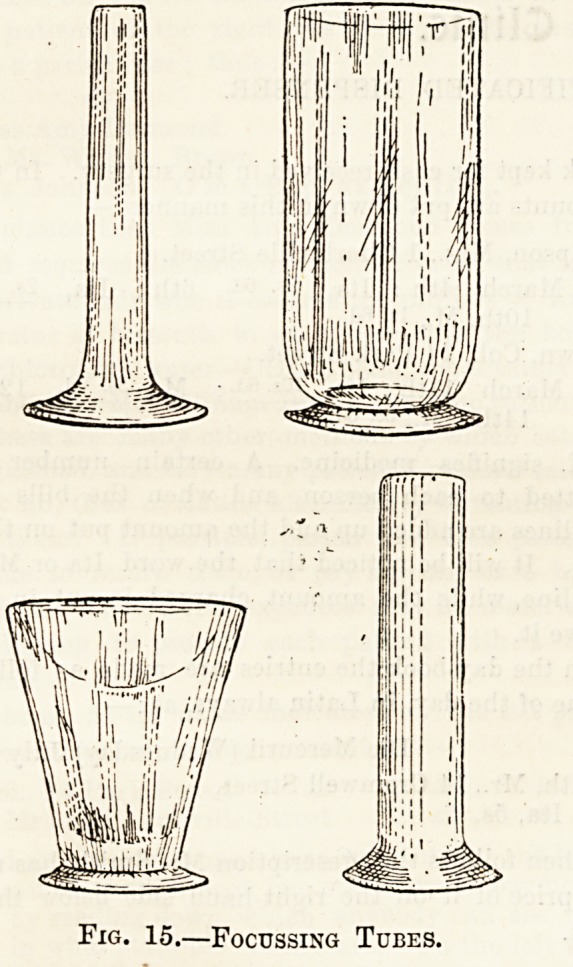


**Fig. 16. f4:**